# Nano-kirigami enabled chiral nano-cilia with enhanced circular dichroism at visible wavelengths

**DOI:** 10.1515/nanoph-2022-0543

**Published:** 2023-01-11

**Authors:** Xing Liu, Qinghua Liang, Xiaochen Zhang, Chang-Yin Ji, Jiafang Li

**Affiliations:** Key Lab of Advanced Optoelectronic Quantum Architecture and Measurement (Ministry of Education), Beijing Key Lab of Nanophotonics & Ultrafine Optoelectronic Systems, and School of Physics, Beijing Institute of Technology, Beijing 100081, China

**Keywords:** chiral nano-cilia, circular dichroism, nanofabrication, nano-kirigami

## Abstract

Nano-kirigami method enables rich diversity of structural geometries that significantly broaden the functionalities of optical micro/nano-devices. However, the methodologies of various nano-kirigami are still limited and as a result, the chiral nano-kirigami structure has yet been pushed to the limit for operation at visible wavelength region. Here, the merits of the various nano-kirigami strategies are comprehensively explored and bio-inspired nano-cilia metasurface with enhanced circular dichroism at visible wavelengths is demonstrated. The stereo chiral nano-cilia metasurface is designed with three-fold rotational symmetry, which exhibits tuneable chiroptical responses when the nano-cilia are deformed to form strong chiral light–matter interactions. By employing electron-beam lithography (EBL) and focused ion beam (FIB) lithography, on-chip nano-cilia metasurfaces are experimentally realized in near-infrared wavelengths region and at visible wavelengths, respectively, successfully validating the giant circular dichroism revealed in simulations. Our work is useful to broaden the existing platform of micro/nano-scale manufacturing and could provide an effective method for the realization of versatile bioinspired nanostructures with profound chiroptical responses.

## 1 Introduction

As an emerging micro/nano-fabrication method, nano-kirigami, including both cutting and deforming processes, has enabled exceptional three-dimensional (3D) nano-geometries through the buckling, bending, rotation and twisting of two-dimensional (2D) nanostructures [1–3]. With the unprecedented 3D nano-geometries and deformable characteristics, the potential applications of nano-kirigami have recently displayed quite profitable in the field of miniaturized devices, such as micro-/nanofabrication field [4, 5], biomedicine [6, 7], microelectromechanical systems (MEMS) [8–10], micro-/nanoscale optical devices [11, 12], etc. Nevertheless, due to the elusive shape change prior to the implementation of 3D transformation, there is always a balance among fabrication resolution, accessibility, adaptability, and compatibility when various optical functionalities are targeted. Meanwhile, although several nano-kirigami strategies have been developed, their merits are still not comprehensively explored and chiral nano-kirigami structures working at visible wavelengths have not yet been realized due to the limited designs.

On the other hand, the design and realization of widely operational bio-inspired nanostructures are of great significance for the bioengineering systems. For example, cilia are generally represented as microscopic hair-like structures in nature, which are early emerged during the natural evolution to provide unicellular organisms with a variety of functions [13]. Recently, inspired by the geometry and property of natural cilia, great interests have been aroused about the fabrication of bioinspired nano-cilia [14–17], the rich sensing features of bioinspired cilia [18, 19], the stimulating movement of cilia under various actuations [20, 21], and so on. Generally, the structure of cilium likes a small hair or flexible rod, whose typical length is between 2 and 15 μm with the diameter of 150–300 nm [22,23] [Figure 2A]. According to recent reports [24–27], various ways of processing cilia and multiple actuating artificial cilia systems have been developed. One important finding is that when cilia are subjected to external stress, they will be not only displaced but also twisted in 3D. The interactions between external physical stimuli and cilia are delivered to electronic or optical signals, where displacements affect the electronic signals and the 3D twisting is largely reflected in the chiroptical responses due to the mirror symmetry breaking. Among the chiroptical responses, circular dichroism (CD) spectrum is widely applied for rapid and accurate detection of chiral molecules for medicinal purpose, biochemistry, and life science [28–31]. Although weak chiroptical response is ubiquitous in natural chiral molecules such as proteins and DNA molecules, chiral plasmonic nanostructures can exhibit a larger CD effect [32–37]. From all the aforementioned aspects, the elaborately designing 3D twisted nano-cilia that consist of plasmonic nanostructures are considered promising to achieve prominent chiroptical properties. However, at nanoscale, it remains challenging to design complex individual ciliary movements and control the coordinated dynamics of chiral ciliary metasurface.

In this study, the merits of the four kinds of nano-kirigami strategies are comprehensively explored and biomimetic nano-cilia metasurface with deformable cilia is studied. The nano-cilia metasurfaces are prepared by electron-beam lithography (EBL) in near-infrared wavelengths and focused ion beam (FIB) at visible wavelengths, respectively, of which the chiroptical responses are comprehensively investigated through both simulations and experiments. The 3D twisted nano-cilia exhibit dramatically enhanced chiroptical response compared to their 2D precursors. Specifically, for the transformed stereo nano-cilia metasurface by nano-kirigami, the transmission difference between two kinds of circularly polarized light (CPL) reaches 80% due to the strong and narrow-band chiroptical response, which can be used as a CPL polarizer. Similarly as the key to the transmission of various mechanical signals in animal hair cilia, the arc length of the nano-cilia also plays a significant role in the engineering of light amplitude and phase modulation. This work not only provides a new study of bioinspired nano-cilia, but also extends the applications of nano-kirigami to the area of biomimetic optical device such as bionic chiral molecular detection and lab-on-a-chip chiral devices.

## 2 Results

### 2.1 Schemes of nano-kirigami

**Figure 1:**
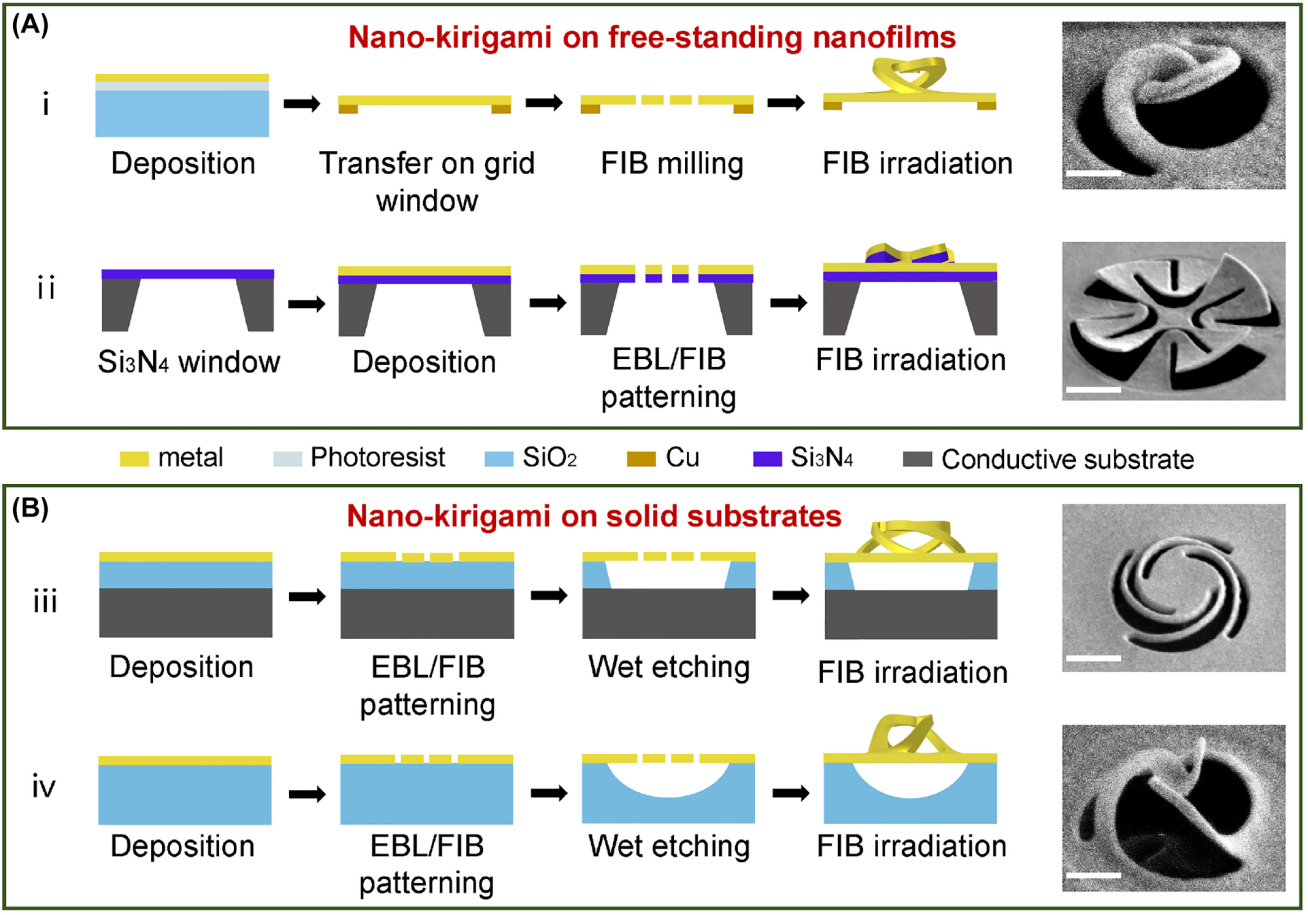
Four schemes of nano-kirigami fabrication process. According to the substrate condition, the nano-kirigami methods can be classified into two types, one on free-standing nanofilms (A) and the other on solid substrates (B). The side-view SEM images of various patterns are displayed in the right side of each scheme. i A home-built method using copper grid to support the free-standing metal membrane for nano-kirigami process. ii Nano-kirigami in commercial Si_3_N_4_ window coated by metallic nanofilms. iii Nano-kirigami methods based on metal/SiO_2_/Si substrates. iv Nano-kirigami methods based on metal/SiO_2_ substrates. Scale bars: 1 μm.

Similar to most kirigami/origami schemes, the early developed strategy of nano-kirigami [8, 38, 39] relied heavily on free-standing nanofilms. For example, in a home-developed nano-kirigami strategy (Figure 1A-i) [38], the free-standing metal films can be obtained by using a copper mesh as the grid window to support the exfoliated metal membrane. The nano-kirigami process can then be readily performed by employing FIB to mill the planar patterns on the single-metal membrane and subsequently deforming the 2D nanostructure into 3D state by low-dose FIB irradiation. The advantage of such scheme is that it can accomplish the 3D nano-kirigami in real-time by simply utilizing one fabrication system in one step. However, it was hard to keep the large-area thin film with desirable flatness due to the involved manual pick-up process. In other words, this scheme was not applicable for large-area nanofabrication (for example several hundred μm^2^).

To address this issue, the second scheme was developed as in Figure 1A-ii [39]. In this scheme, a commercial film window (for example silicon nitride nanofilm) was employed, on which the metallic nanofilms can be deposited for following nanofabrication. In such a case, the multilayer nanofilms can be precisely patterned by EBL or FIB technique and deformed into 3D nano-kirigami structures. With this strategy, the nanofilms can be kept very flat and the nano-kirigami method can be implemented in large scale. However, the structures are no longer formed by single material and if the film window is as wide as in millimeter, the fabricated nanostructures will be very fragile, which are not desirable for device integration. Actually, integration is a main challenge faced by all kinds of kirigami structures fabricated on free-standing substrates.

To avoid the fragility induced by large-area free-standing nanofilms, a local suspending strategy was explored. As illustrated in Figure 1B-iii [8], a solid multilayer film consisting of top metal, middle oxide layer, and bottom conductive layer was used for the 2D patterning. Subsequently, a wet etching process was employed to locally remove the middle oxide layer exactly beneath the lithographed 2D patterns. In such a case, the 2D patterns were locally suspended from the solid substrates, which were strongly supported by the unetched pillar areas. Under FIB irradiation, the locally suspended 2D patterns can then be deformed into 3D by the traditional nano-kirigami principle. In such a case, the nano-kirigami structures can be fabricated on large-scale solid substrate and the rigidity of final deformed nanostructures was greatly improved. Moreover, since the bottom layer was conductive, introduction of voltage between the top and bottom substrate can generate electrostatic force, with which the nano-kirigami structures can be dynamically tuned via the externally applied voltage. Although this scheme solved the challenges of large-scale flatness and structural rigidity, such scheme was not applicable for transmission-type characteristics since the conductive substrate was too thick to optically transparent.

Here, to address the above challenges and meet the requirement of transmission-type characterization, a novel strategy based on solid metal/SiO_2_ layer structure is proposed for the first time in Figure 1B-iv. In this scheme, the transmission-type nano-kirigami can be implemented in large-scale area with extreme flatness, which is compatible with traditional nanofabrication systems for further devices applications. For example, this technique is best suited to manufacture the nano-cilia, which needs to measure the transmission spectra to characterize the deformation-dependent optical chirality. Therefore, in the following study, we will mainly concentrate on this fabrication strategy.

### 2.2 Design and fabrication schemes of chiral nano-cilia

**Figure 2:**
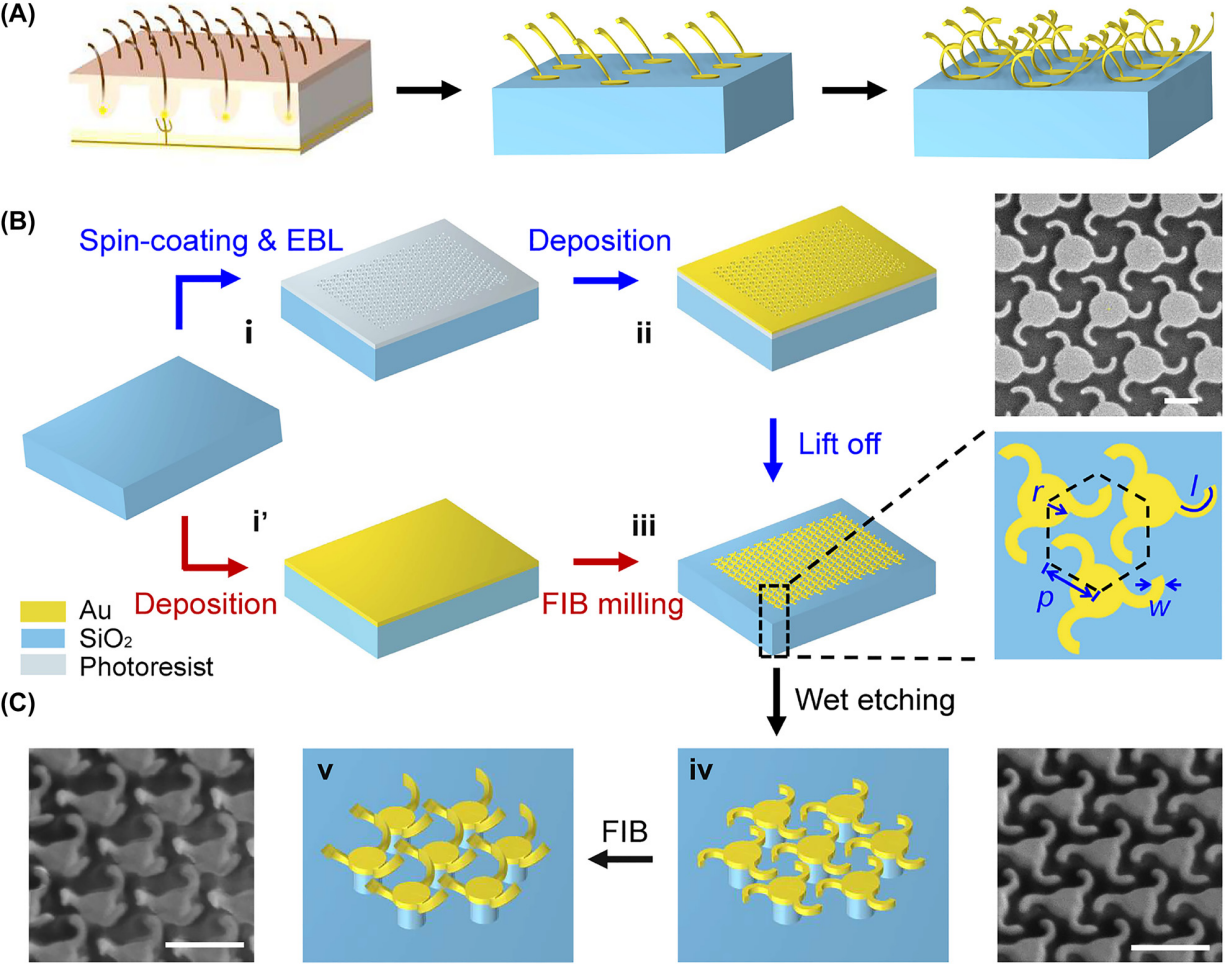
Schematic illustration of the fabrication process of the chiral nano-cilia arrays. (A) Left, schematic illustration of the hair cells and human skin. Middle and right, unidirectional and chiral nano-cilia arrays. (B) Two schemes for the 2D patterning on silica substrate with EBL and FIB, respectively. (C) The stereo bioinspired nano-cilia are obtained after wet-etching and subsequent deformation by low-dose FIB. Corresponding structural models and SEM images of the nano-cilia processed by different techniques and in different steps are shown for illustration. Scale bars: 500 nm.

Inspired by nature, the artificial nano-cilia have been recently developed, in which the vertical movement or sensing feature is studied under the influence of various stimuli such as electrostatic forces [27, 40, 41], magnetic fields [42, 43], pressure [44, 45], light [46, 47],etc. However, in nature, cilia grow in diverse directions and when subjected to external stress they are not only displaced but also twisted in complex formation. Based on this feature, we propose nanoscale cilia metasurface with twisting cilia configurations and chiral features according to the shape of the natural cilia and their sensing characteristics, as illustrated in Figure 2A. Such right-handed twisted nano-cilia metasurface can be fabricated by EBL or FIB, as shown in Figure 2B. Each unit cell contains three cilia which is attached to the central Au disk at one end.

In EBL process, to start with, in Figure 2B-i, the silica substrate is coated with 120-nm-thick electron resist (poly-(methyl methacrylate) (PMMA), AR-P-672.02) and baked at 180 °C for 60 s. Subsequently, the designed nano-patterns are exposed by E-beam in a FIB/EBL dual-beam system (Helios G4 UC). In the following step (Figure 2B-ii), a bilayer of 5-nm-thick chromium (Cr) and 60-nm-thick gold (Au) are sequentially deposited onto the sample by using an E-beam evaporation system. After the lift-off process, the 2D nano-cilia are finally obtained in Figure 2B-iii, as the SEM image shown in the upright of Figure 2B.

The FIB fabrication process is more straightforward from step i’ to step iii, in which the nano-cilia pattern can be directly formed by using the high-dose FIB. As shown in Figure 2B, the three-arm metallic patterns with a width of *w* = 60 nm are fabricated in a hexagonal lattice, as enclosed by the black dashed lines. The planar unit with periodicity of *p* consists of a cylinder to support three curved cantilevers, in which the arc length *l* of the nephroid curve and the radius *r* = 130 nm of the cylinder are defined.

After the 2D patterning, as shown in Figure 2C, the nano-cilia are released from the substrate and the center is supported by the 300-nm-thick SiO_2_ pillars through an accurate wet-etching process (side-view SEM image of the nano-cilia after wet-etching is shown in the step iv). To simulate the stress response of the chiral nano-cilia, the low-dose FIB irradiation is employed to induce tensile stress for final deformation, causing the originally 2D planar nanostructure to be twisted and vertical displaced around the central cylinder, as schematically illustrated in Figure 2C-v. In such a case, the fabricated nano-kirigami provides a novel method to obtain nanoscale cilia, whose typical length can be designed according to the biological cilia.

### 2.3 Simulated chiroptical response of the nano-cilia

**Figure 3:**
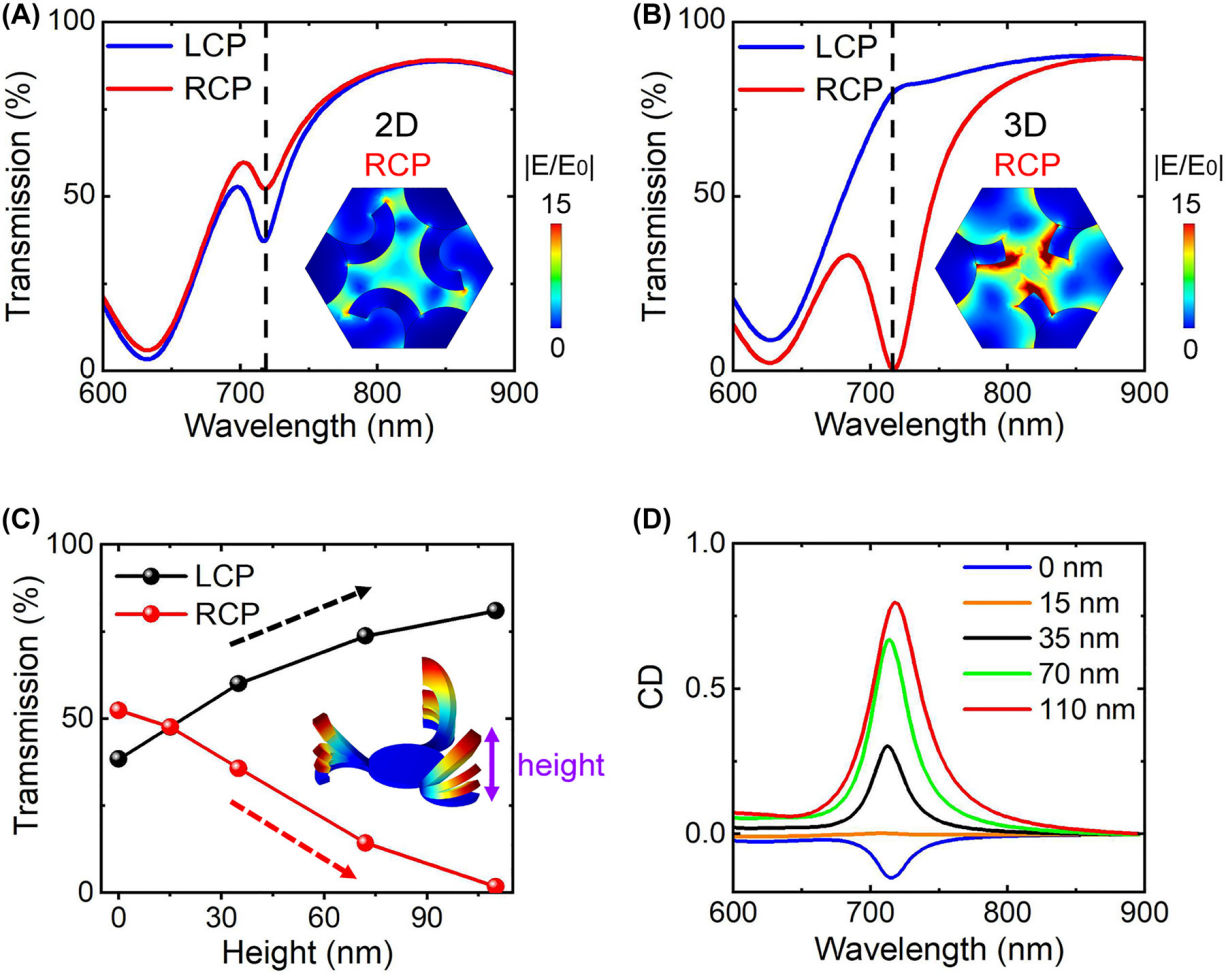
Chiral response of the exotic nano-cilia in calculations. (A and B) Calculated transmission spectra of (A) 2D (with height of 0 nm) and (B) 3D deformed (with height of 110 nm) nano-cilia under excitations of RCP and LCP light, where *p* = 500 nm. Inset, normal electric field distributions of the 2D and 3D nano-cilia in the plane of *z* = 0.03 μm (in the middle of nano-cilia structures) at the resonant wavelength under RCP excitation. Strongly electric fields are generated among the arms of the deformed nano-cilia. (C) Calculated transmission intensity of 2D and 3D deformed nano-cilia under excitation of RCP and LCP light with wavelength of 716 nm. Inset, illustration of the simulated structures with corresponding deformation height. (D) Calculated CD spectra of the nano-cilia with different deformation height as noted. After deformation, the initial CD dip changes into a strong and narrow CD peak under deformation height of 110 nm, which can be used as an effective chiral polarization switch since the RCP transmission reaches 0 at wavelength 716 nm (Figure 3B).

The chiroptical response of the proposed bionic nano-cilia with different heights are characterized by studying the transmission of the CPL. For the 2D planar nano-cilia with *p* = 500 nm, the simulated transmission spectra under left- and right-handed circularly polarized (LCP and RCP) light incidence show similar features, as plotted in Figure 3A. A shallow plasmonic resonance dip appears at 716 nm for both LCP and RCP excitations, where the transmission amplitude shows a little difference. According to the principles of symmetry and reciprocity [48, 49], the coupling between the three-fold-rotational-symmetry (C3) structure and CPL light can be described by the S-matrix equation, in which the transmitted light does not generate the cross-polarized components and the reflection components of RCP and LCP incident are the same. Meanwhile, the transmission spectra are the same for both illumination along +*z* and −*z* directions (results not shown). Thus, for the chiral nano-cilia, the circular dichroism [49] can be determined as CD = *T*_LCP_ – *T*_RCP_, where *T*_LCP_ (*T*_RCP_) is the total transmission under the LCP (RCP) excitation. Here, the proposed 2D nano-cilia exhibit a small negative CD dip (blue curve in Figure 3D) due to the existence of the silica pillar substrate that breaks the mirror symmetry, the difference of which can also be seen from the electric field distributions (Figure S3 of Supplementary Information). Such a substrate effect can be verified by the fact that the difference in transmission for RCP and LCP light disappears (CD = 0) when the silica pillar substrate is replaced by air (Figure S1 of Supplementary Information).

The structural asymmetry with respect to the *xy* plane can be further reinforced by applying stress on the 2D nano-cilia, which causes the vertical deformation and increases the height from 0 to 110 nm. Although the deformation height is small, the RCP transmission spectrum of the deformed nano-cilia changes dramatically and RCP transmission amplitude is significantly smaller than that of the LCP at the resonance wavelength of 716 nm. This is due to the fact that at the resonance wavelength, the LCP transmission increases with the deformation height, while the RCP transmission decreases, as plotted in Figure 3C (see more details in SI Figure S2). As a result, a prominent CD dip appears at wavelength 716 nm for the 2D pattern, which evolves into a peak with value of as high as 0.8 (the red curve in Figure 3D). Interestingly, it is found the CD reaches nearly zero for the 3D nano-cilia with a height of 15 nm. This is caused by the handedness-dependent excitation of electromagnetic multipolar modes (Figure S3 of Supplementary Information), the combination of which determines the value of CD. As a result, the 2D structure with *h* = 0 nm could show a tiny CD but a 3D chiral structure with *h* = 15 nm shows zero CD at specific wavelengths, as revealed in Figure 3D. Therefore, the CD of nano-kirigami structure is highly associated with the special deformed geometries and the CD amplitude exhibits a positive correlation with the height of nano-cilia. The transmission of the deformed 3D nano-cilia can even reach zero under RCP excitation, which can be utilized as an effective chiral polarization switch.

In order to reveal the physical mechanism of the CD effect upon 3D deformations, the normal electric field intensity distributions of the 2D and 3D chiral nano-cilia are simulated and plotted at resonance wavelengths (SI Figure S3). In the 2D case, under LCP excitation, the position of the electric field hot spots is located around the tip of the planar bionic nano-cilia at 716 nm. In contrast, the weak interaction between the cilia and the adjacent center parts occur when the RCP light illuminates on the 2D nano-cilia (inset of Figure 3A). Such a weak interaction dramatically changes to strong interaction when the nano-cilia are deformed into 3D geometries. As shown in the inset of Figure 3B, under RCP illumination, the E-field hotspots are found strongly localized at the tip of the nano-cilia and the strong interaction is formed among the neighboring nano-cilia. The scattering power spectra of 3D nano-cilia (SI Figure S3) indicate that the handedness-dependent excitation of electric dipole (ED) mode plays a major role.

### 2.4 Experimental chiroptical properties of nano-cilia metasurfaces

**Figure 4:**
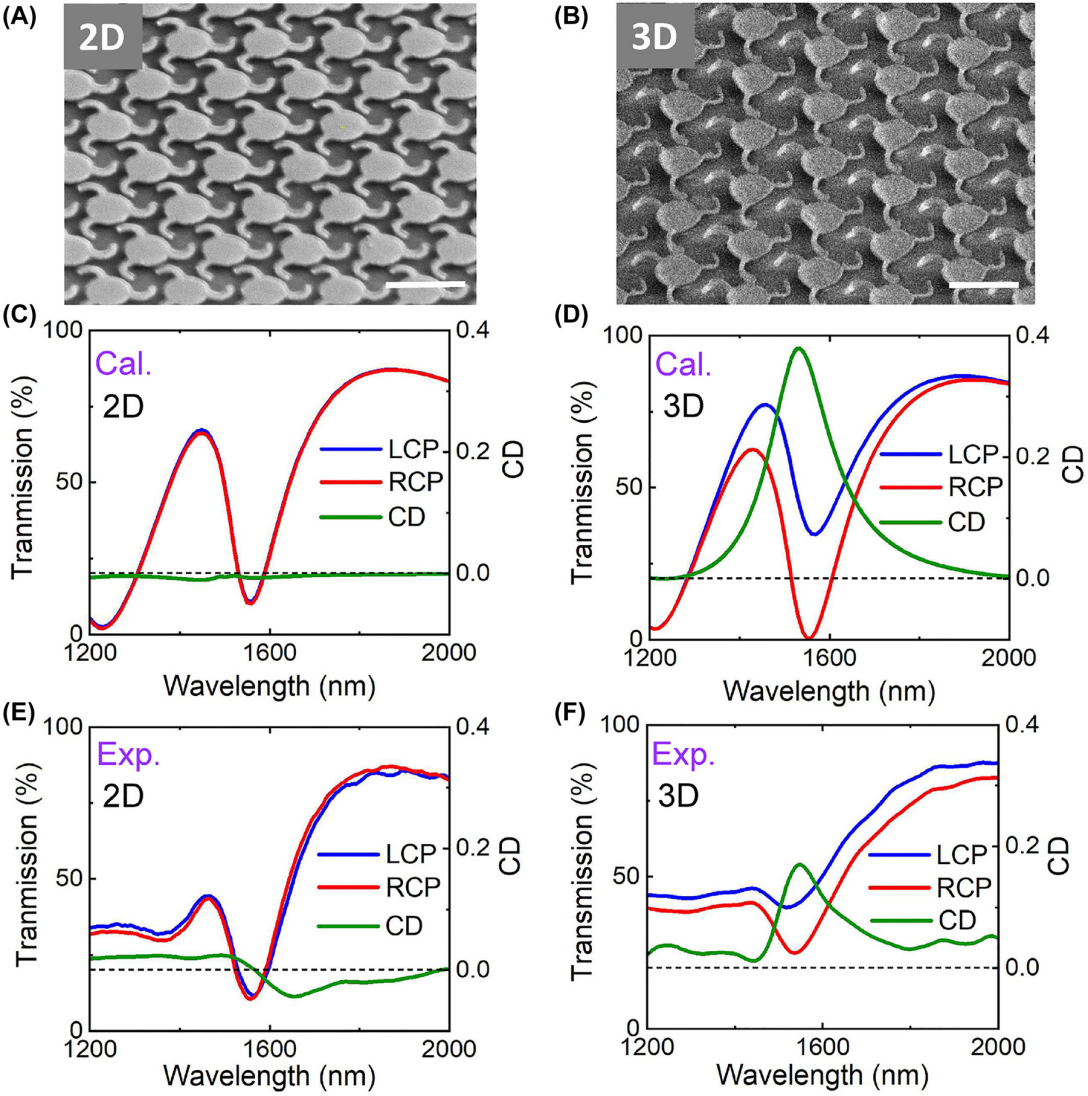
Experimental demonstrations of the nano-cilia metasurface in near-infrared wavelength region by EBL process. (A and B) Side-view SEM images of the fabricated 2D and 3D deformed nano-cilia, respectively, with a periodicity of 1.2 μm. Scale bars: 1.2 μm. (C and D) Simulated and (E and F) measured transmission spectra of the 2D and 3D nano-cilia, respectively, under LCP or RCP light excitation. Corresponding CD spectra are shown in the right side of each spectrum.

To experimentally realize the proposed designs, the nano-cilia are firstly fabricated by using the EBL scheme in Figure 2B. As shown by the side-view SEM images in Figure 4A and B, the 2D nano-cilia precursors and 3D deformed nano-cilia are successfully realized (see more details in Supporting Information Figure S4). The overall size of the fabricated array is 25 µm × 25 µm, in which *p* is 1.2 μm. The fabricated nano-cilia metasurface is further characterized by using a home-built microscopy system [50]. Figure 4E and F plot the optical measurements of 2D planar and 3D deformed nano-cilia, respectively, in which the dips of LCP and RCP transmission spectra show good agreement with the simulations results (the calculated curves in Figure 4C and D) in near-infrared wavelengths. The small deviations at wavelengths from 1200 to 1400 nm are caused by the fabrication imperfections, which induce unwanted scatterings that influence the spectra more seriously at short wavelength region. Moreover, the 2D pattern shows nearly identical transmission spectra under LCP and RCP excitations, indicating almost no CD (green line in Figure 4E). In comparison, the 3D deformed nano-cilia exhibit a clear CD peak, as the green curve shown in Figure 4F, clearly verifying the numerical observations.

**Figure 5:**
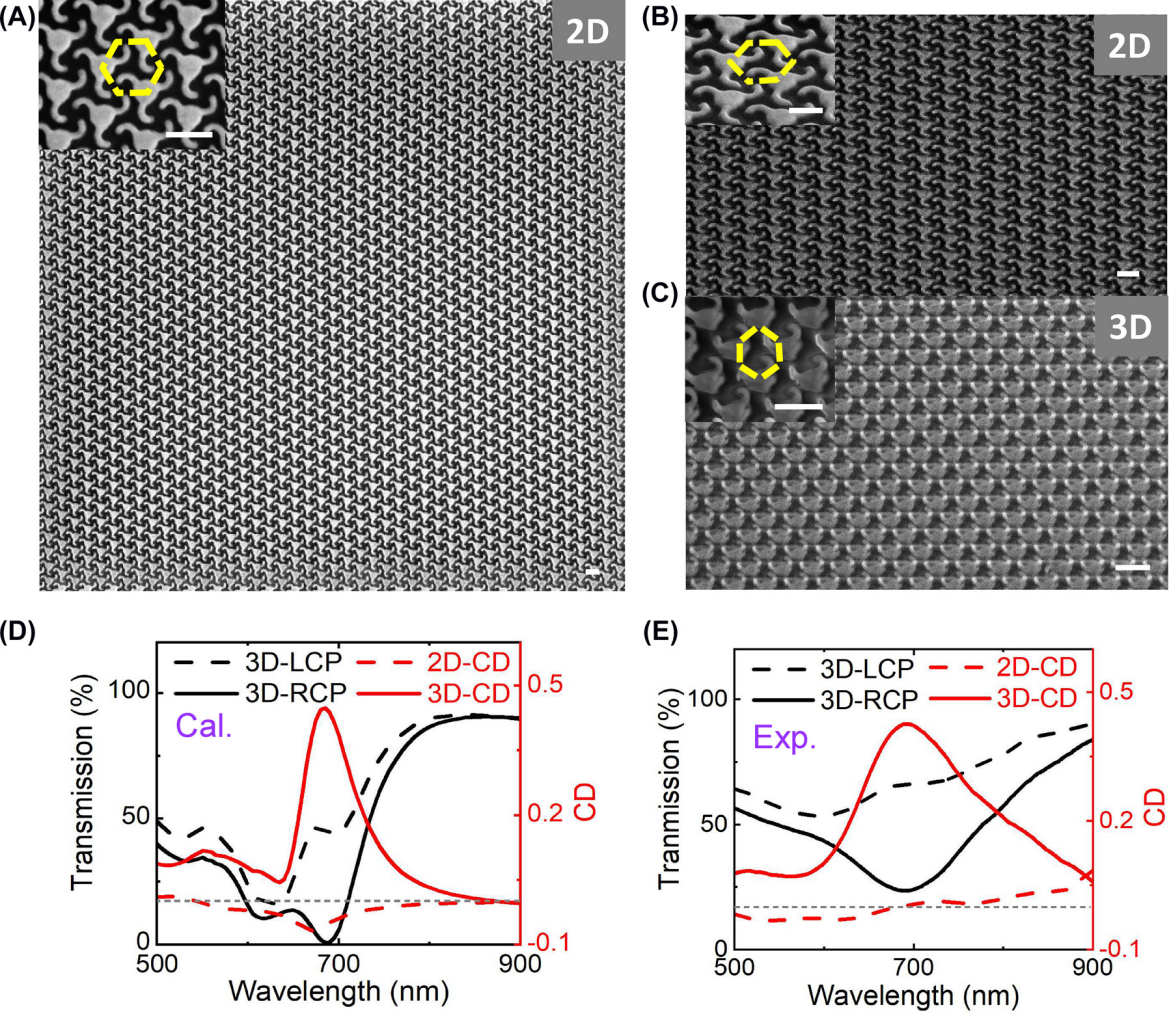
Experimental demonstrations of the nano-cilia metasurface at visible wavelength region by FIB process. (A–C) Top-view and side-view SEM images of 2D and 3D deformed nano-cilia metasurfaces with a period of 500 nm. Scale bars: 500 nm. (D) Left, simulated transmission spectra of 3D deformed nano-cilia under LCP and RCP excitation. Right, calculated CD spectra of the 2D and 3D nano-cilia as noted. (E) The corresponding measured results of D.

To realize the chiral nanostructures operating in visible wavelengths, reducing the size of nano-kirigami is of great significance. However, previous nano-kirigami strategies are based on the close-loop designs, making it challenging to shrink the size of the nanostructures. Here, by employing the open-loop designs, the proposed nano-cilia can be fabricated in much small scale and the chiroptical responses can thus be pushed into visible wavelength region. As shown in Figure 5A–C, the FIB milling is utilized to directly fabricate the 2D nano-pattern and subsequent global FIB irradiation is employed to continuously deform the nano-cilia into 3D geometries (see continuous deformation in Supporting Information Video). The calculated and measured LCP/RCP transmission spectra of the 3D deformed nano-cilia, as well as the CD spectra for 2D and 3D nano-cilia, are plotted in Figure 5D and E, respectively (the calculated and measured LCP/RCP transmission spectra of the 2D structures are shown in Figure 5 of Supplementary Information). It can be seen that both the calculated and measured CD responses of the 2D structure are very weak and no peak are observed, which is caused by the fact that at the dip wavelength, the pillar height is about *λ*/5 and the substrate effect is negligible. After the deformations, the 3D nano-cilia metasurface shows a clear CD peak at wavelength 690 nm, as plotted by the solid red line in Figure 5E. Such an experimental observation is consistent with the simulation results in Figure 5D, although the CD peak width is broad due to the material loss possibly induced by the implantation of Ga^+^ ions. Nevertheless, this is the first demonstration of chiral nano-kirigami structure that operates at visible wavelengths, which could expand the diversity of studying biomimetic cilia and other on-chip bioinspired chiral optical devices.

## 3 Conclusions

In summary, we have demonstrated an efficient nano-kirigami method to fabricate nano-cilia metasurface with 3D twisting features. Various nano-kirigami strategies, as well as their merits and disadvantages, are comprehensively explored. The nano-cilia metasurface consist of the cilium-inspired cantilevers and the center plates supported by silica pillars. As the arms of the cilia cell are deformed from the planar to stereo geometry, dramatic changes in CD responses occur and thereby a sharp CD peak with amplitude of 0.8 is observed due to strong interaction among adjacent nano-cilia. By employing EBL and FIB associated fabrication schemes, on-chip nano-cilia with stereo deformations have been experimentally realized in near-infrared wavelength region and at visible wavelengths, respectively. For the first time, the chiral nano-kirigami structures are experimentally demonstrated at visible wavelengths, in which the deformation breaks the mirror symmetry and thus induces strong CD response. It should be mentioned that the optical responses of cilia with different motion directions or orientation angles (with the same height) can also affect the chiral optical spectrum, and the arc lengths of the nano-cilia can be used for the polarization selection effect towards the engineering of transmission amplitude and gradient phase (see SI Figures 6 and 7). Although the designed nano-cilia are still simple compared to the biological cilia, the handedness-dependent transmission sensitivity to cilia length at specific wavelengths provides a novel chiral sensing mechanism, which deserves future optimization through the topographic designs. Therefore, this work not only provides useful strategies for various nano-kirigami methods according to specific purposes, but also broadens the applications of nano-kirigami structures in the area of chiroptics and bioinspired devices, for example the chiral nano-cilia metasurfaces for light control and microfluidic manipulation [47, 51].

## Supplementary Material

Supplementary Material Details

Supplementary Material Details
