# Supplementary figures and images for: Nano-kirigami enabled chiral nano-cilia with enhanced circular dichroism at visible wavelengths
Source: Nanophotonics. 2023 Jan 11;12(8):1459–68. doi: 10.1515/nanoph-2022-0543 (PMC11502046; doi:10.1515/nanoph-2022-0543)

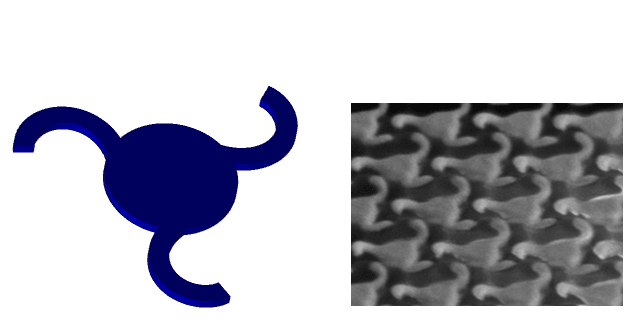

Supplement: Supplementary file 1 — Supplementary Material Details [file j_nanoph-2022-0543_suppl_001.gif]
