# Supplementary material for: Nano-kirigami enabled chiral nano-cilia with enhanced circular dichroism at visible wavelengths
Source: Nanophotonics. 2023 Jan 11;12(8):1459–68. doi: 10.1515/nanoph-2022-0543 (PMC11502046; doi:10.1515/nanoph-2022-0543)
Supplement: Supplementary file 2 — Supplementary Material Details [file j_nanoph-2022-0543_suppl_002.pdf]

Xing Liu[1], Qinghua Liang[1], Xiaochen Zhang[1], Chang-Yin Ji[1], Jiafang Li*[1]
[1] *Key Lab of Advanced Optoelectronic Quantum Architecture and Measurement (Ministry of Education), Beijing Key Lab of Nanophotonics & Ultrafine Optoelectronic Systems, and School of Physics, Beijing Institute of Technology, Beijing 100081, China*
* jiafangli@bit.edu.cn

Nano-kirigami enabled chiral nano-cilia with enhanced circular dichroism at visible wavelengths

Supplementary Note 1:

Numerical simulations. The numerical simulations are conducted by the finite element software COMSOL. Based on the bilayer stress distribution model [1], the structural transformation from the planar sample into the deformed nano-kirigami is implemented by mechanical simulations. The transmission spectra and electromagnetic field distributions are calculated using circularly polarized light incident along the negative *z*-axis. In the *x*–*y* plane, periodic boundary conditions are applied to the corresponding unit cell and the perfectly matched layer boundary condition is employed for the *z* direction.

Sample Fabrications. The designed 2D nano-cilia are fabricated by a FIB/EBL dual-beam system (Helios G4 UC). For FIB milling, a high dosage of >600 pC/μm^2 is employed to cut the 2D nano-patterns and the deformed nano-cilia are realized by globally irradiating the entire metasurface with a relative low dose of 10-40 pC/μm^2. The tensile stress causing the deformation of nano-kirigami is induced by the interactions between the high-energy gallium ions (Ga^+) and the gold nano-film, in which partial gold atoms are sputter from the film surface with vacancies behind, partial Ga^+ are implanted into the film, partial gold atoms are dislocated, and some gold and gallium atoms are redeposited (see more details in [2, 3]).

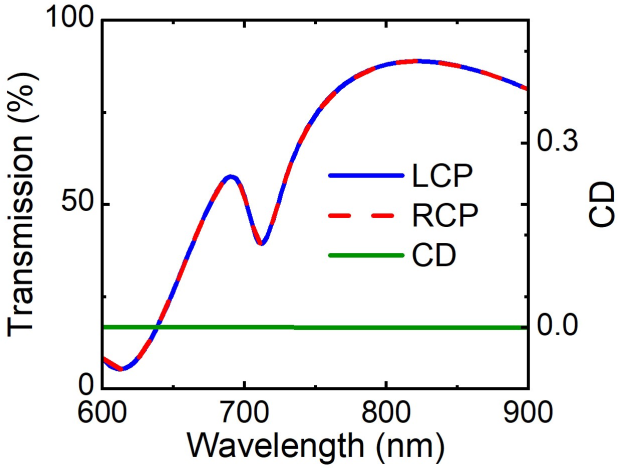

Figure S1: The transmission spectra of the nano-cilia without SiO_2 substrate (in air) under excitation of RCP and LCP light, which show no difference. Corresponding CD spectrum is shown in the right side of figure.

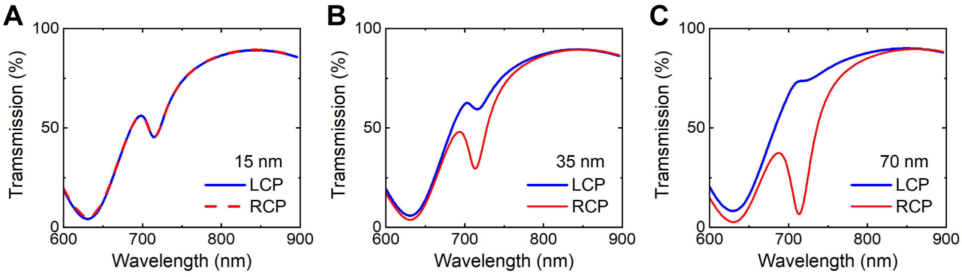

Figure S2: Calculated transmissions spectra of the nano-cilia with different deformation heights of 15 **nm** **A**, 35 nm **B** and 70 nm **C** as noted.

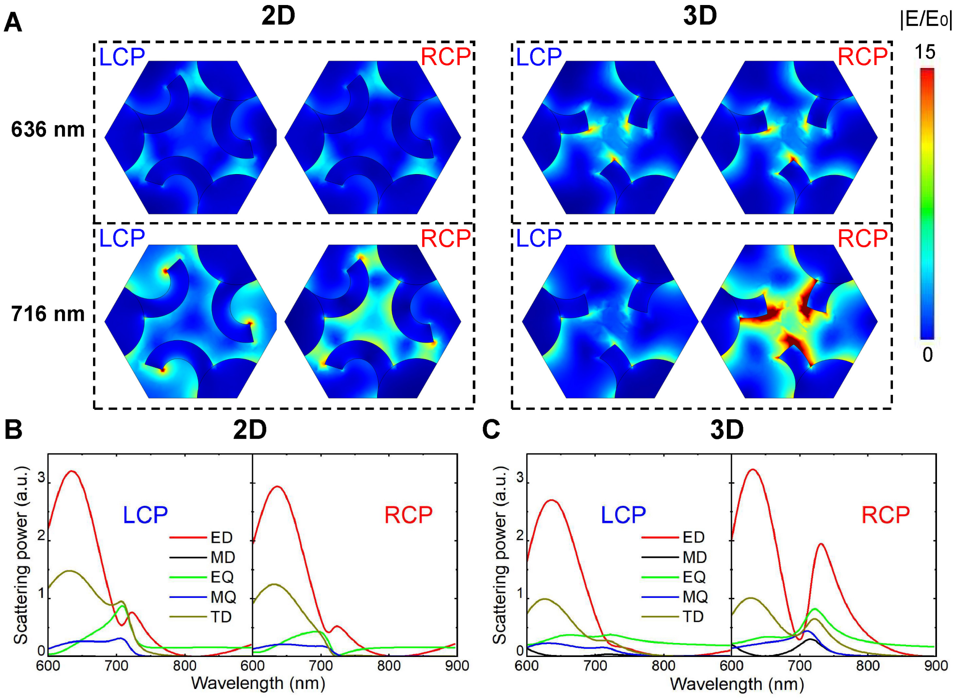

Figure S3: A Normal electric field distributions and **B**, **C** scattering power spectra of various multipole modes generated from the 2D and 3D nano-cilia under RCP and LCP light, respectively. ED: electric dipole; MD: magnetic dipole; EQ: electric quadruple; MQ: magnetic quadrupole, TD: electric toroidal dipole.

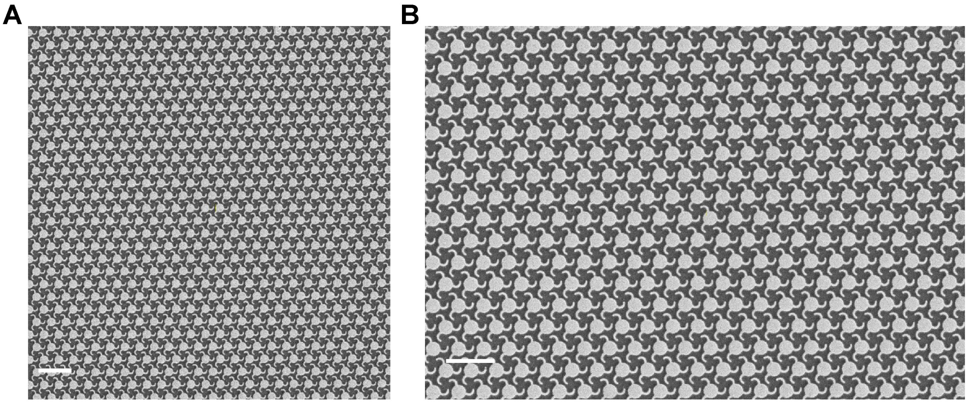

Figure S4: A, **B** Top-view SEM images of chiral nano-cilia metasurface processed by EBL after wet-etching. Scale bars: 2.4 μm.

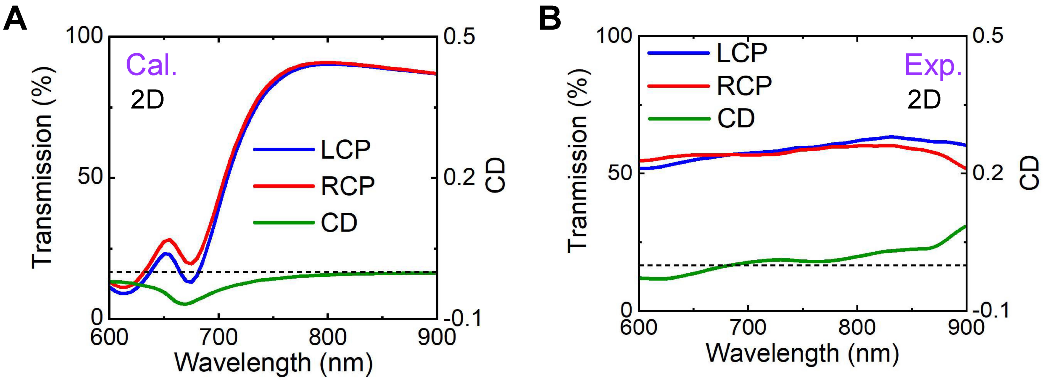

Figure S5: A Simulated and **B** measured transmission and CD spectra of the 2D nano-cilia metasurface by FIB process.

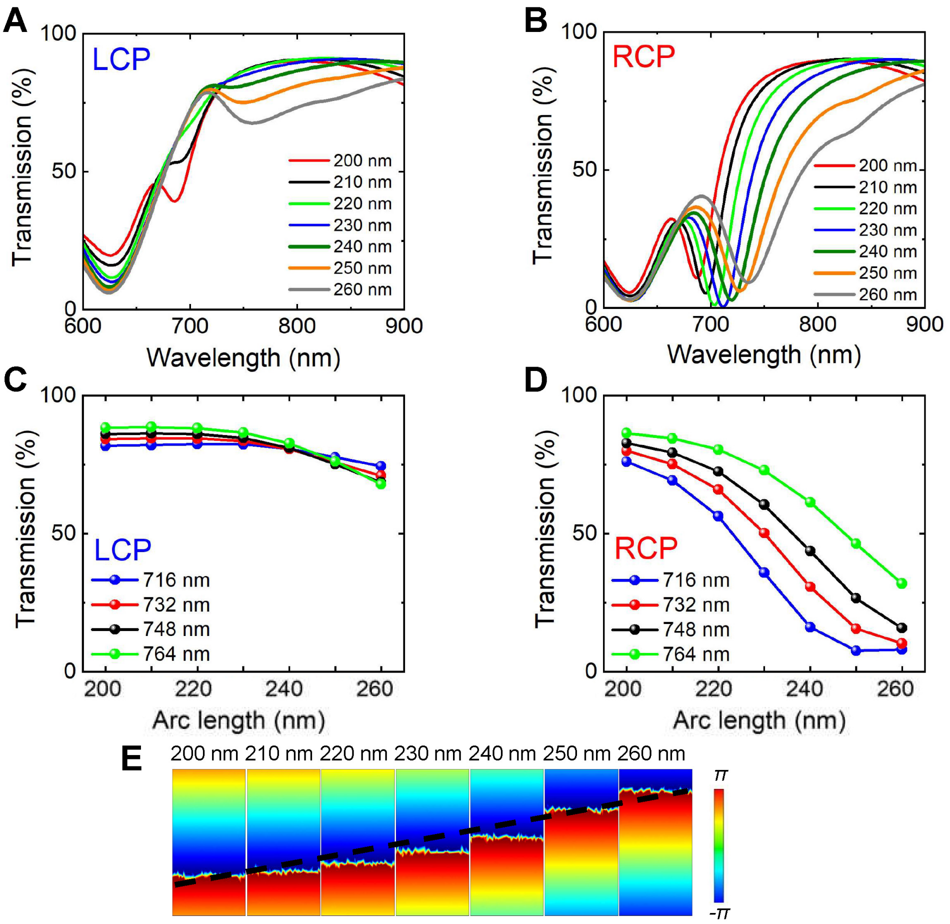

Figure S6: A, **B** Transmission spectra of 3D nano-cilia with different arc lengths (keeping the width unchanged) under incident LCP and RCP wave, respectively. Intensity of LCP **C** and RCP **D** transmission at wavelengths near the second resonance wavelength for the stereo nano-structures. **E** Simulated phase distribution obtained under RCP incidence at 716 nm wavelength for nano-cilia with different lengths as noted. It can be seen that by choosing nano-cilia with different arc length from 210 to 260 nm, the transmission phase can be obtained in the range of [-π, +π]. With this capability, the nanostructures with different arc lengths can be deliberately selected and arranged in certain manner to manipulate the wave front of the incident light, which is promising in the applications of emerging metasurfaces.

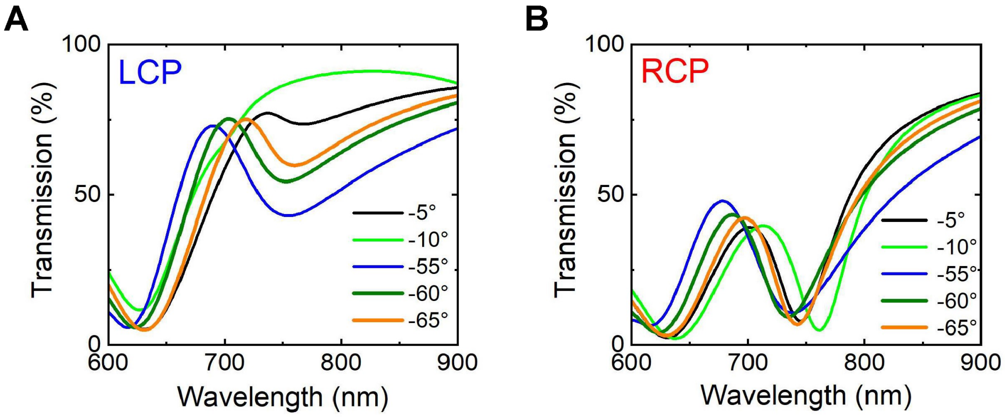

Figure S7: A, **B** Simulated transmission spectra of RCP and LCP incidence for the 3D stereo nano-cilia with various rotation angles.

Supplementary References

[1] Z. Liu, H. Du, J. Li, et al., "Nano-kirigami with giant optical chirality," *Science Advances*, vol. 4, no. 7, pp. eaat4436, 2018. https://doi.org/10.1126/sciadv.aat4436

[2] J. Li and Z. Liu, "Focused-ion-beam-based nano-kirigami: From art to photonics," *Nanophotonics*, vol. 7, no. 10, pp. 1637-1650, 2018. https://doi.org/10.1515/nanoph-2018-0117

[3] Z. Liu, H. Du, Z. Y. Li, et al., "Invited Article: Nano-kirigami metasurfaces by focused-ion-beam induced close-loop transformation," *APL Photonics*, vol. 3, no. 10, pp. 100803, 2018. https://doi.org/10.1063/1.5043065
